# Microendoscopic Surgery for Degenerative Disorders of the Cervical and Lumbar Spine: The Influence of the Tubular Workspace on Instrument Angulation, Clinical Outcome, Complications, and Reoperation Rates

**DOI:** 10.3390/jpm13060912

**Published:** 2023-05-30

**Authors:** Joachim M. Oertel, Benedikt W. Burkhardt

**Affiliations:** 1 Klinik für Neurochirurgie, Universität des Saarlandes, Kirrbergerstrasse 100, 66421 Homburg-Saar, Germany; oertelj@freenet.de; 2 Wirbelsäulenzentrum/Spine Center—WSC, Klinik Hirslanden, Witellikerstrasse 40, 8032 Zurich, Switzerland

**Keywords:** cervical spine, complication, clinical outcome, degenerative disc disease, endoscopy, instrument angulation, microendoscopic, lumbar spine, reoperation

## Abstract

Background: Long-term clinical outcomes with microendoscopic spine surgery (MESS) are poorly investigated. The effect of instrument angulation on clinical outcomes has yet to be assessed. Methods: A total of 229 consecutive patients operated on via two MESS systems were analyzed. Instrument angulation for both MESS systems, which differ from each other regarding the working space for instruments, was assessed using a computer model. Patients’ charts and endoscopic video recordings were reviewed to determine clinical outcomes, complications, and revision surgery rates. At a minimum follow-up of two years, clinical outcomes were assessed employing the Neck Disability Index (NDI) and Oswestry Disability Index (ODI). Results: A total of 52 posterior cervical foraminotomies (PCF) and 177 lumbar decompression procedures were performed. The mean follow-up was six years (range 2–9 years). At the final follow-up, 69% of cervical and 76% of lumbar patients had no radicular pain. The mean NDI was 10%, and the mean ODI was 12%. PCF resulted in excellent clinical outcomes in 80% of cases and 87% of lumbar procedures. Recurrent disc herniations occurred in 7.7% of patients. The surgical time and repeated procedure rate were significantly lower for the MESS system with increased working space, whereas the clinical outcome and rate of complication were similar. Conclusions: MESS achieves high success rates for treating degenerative spinal disorders in the long term. Increased instrument angulation improves access to the compressive pathology and lowers the surgical time and repeated procedure rate.

## 1. Introduction

Degenerative disorders of the cervical and lumbar affect about 5% of the population in Western civilization, with a rising prevalence due to aging [1]. Arm and leg pain is the cause of severe morbidity and a significant economic burden [2]. In the 1970s, G. Yasargil and W. Caspar independently published their technique of microsurgical lumbar discectomy, which is still considered the “gold standard” for treating lumbar disc herniation (LDH) [3,4]. In 1940, Scoville and Frykholm reported their experience using a posterior cervical foraminotomy to treat cervical radiculopathy [5,6]. Over the past decades, multiple studies have demonstrated that those surgical procedures provide excellent long-term results for treating patients who have failed conservative treatment [7,8,9].

The idea of minimally invasive spine surgery (MISS) dates back to the 1970s [10]. Since then, MISS techniques have been further developed and evolved concerning visualization of the surgical field [11,12]. The significant advantage compared to an open procedure is the reduction of soft tissue and muscle trauma during the approach [13,14]. Less blood loss, lower postoperative opioid use, a shorter hospital stay, and a lower postoperative infection rate have been reported [15,16].

Concerns have been raised that MISS might increase the risk of perioperative complications, inadequate decompression, and recurrent LDH [8,17,18,19]. Critics see the cause of these events in poor image quality, the need for advanced technical expertise, and the need for specialized equipment that might differ from standard microsurgical instruments.

In this article, the authors report their experiences using improved microendoscopic spine surgery (MESS) systems to treat degenerative cervical and lumbar disorders. In this single-center study with a mean follow-up of six years, the authors assessed the long-term clinical outcome, complication, and revision surgery rates when employing modern MESS systems with improved instrument angulation and, thus, surgical access to the compressive pathology.

## 2. Materials and Methods

### 2.1. Study Design, Patients, Exclusion/Inclusion Criteria

A prospectively collected database of endoscopic spine procedures was reviewed retrospectively. The surgical report and the endoscopic video recording were analyzed to identify all patients who underwent an endoscopic procedure for the treatment of degenerative disorders of either the cervical or lumbar spine from January 2011 to March 2018.

#### 2.1.1. Inclusion Criteria for Further Evaluation Were as Follows

(1) Complete set of preoperative and postoperative patient records as well as outpatient visit notes;
(2) The video recording of the endoscopic procedure performed using a MESS system;
(3) Paramedian approach to the cervical or lumbar spine.

#### 2.1.2. Exclusion Criteria Were as Follows

(1) Endoscopic procedure performed with a system other than a microendoscopic system (MESS), i.e., a fullendoscopic spine surgery system);
(2) Endoscopic surgery for a diagnosis other than a degenerative disorder;
(3) Endoscopic procedure performed at the thoracic spine;
(4) Other than a paramedian (i.e., fullendoscopic or microendoscopic posterolateral, fullendoscopic transforaminal) approach to the spine.

### 2.2. Microendoscopic Spine System (MESS)

**Figure 1:**
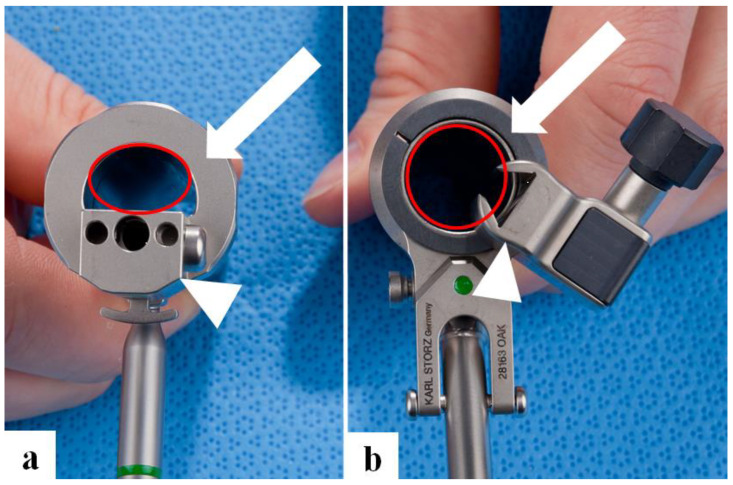
Shown is the EasyGO system view from above, illustrating the available workspace (red circled area) within the tubular retractor for the instrument workspace. The first-generation EasyGO™ system (**a**) is shown in the **left** panel, and the second in the **right** panel (**b**). The retractor system has a tubular retractor holder (white arrow) and another holder to insert the endoscope (white arrowhead).

For the MESS surgeries, the first and second-generation EasyGO^®^ system (KARL STORZ GmbH & Co. KG, Tuttlingen, Germany) was used to perform all cervical and lumbar procedures. The first generation of the EasyGO system was replaced by the second generation due to the refinement of the endoscope holder. The design of the endoscope holder changed from the first to the second generation of EasyGO™ (Figure 1). Once the second generation of the EasyGO system was introduced, all procedures were performed using this MESS system. The outer diameter of the tubular retractor did not vary between both generations. Three different diameters of the tubular retractor are available, ranging from 15 mm (orange color code), 19 mm (green color code), and 23 mm (black color code). The tubular retractor is available in 40 mm, 70 mm, and 90 mm lengths. The ring-shaped endoscope holder of the second generation can be rotated up to 270° in any direction and fixed with a locking screw. Visualization of the surgical field is obtained using an endoscope with a 25° viewing angle. The length of the endoscope matches the length of the tubular retractors. Hence, there are three endoscopes in the EasyGO™ set [12]. Due to the new design of the endoscope holder, the instrument work area has increased by 23.7% for the 15 mm retractor, 18.2% for the 19 mm retractor, and 13.8% for the 23 mm retractor (Figure 1).

### 2.3. Surgical Technique

The cervical spine surgeries were performed under general anesthesia with the patient in a prone position. The head was inclined, elevated, and fixed in Gardner-Wells tongues in the military position. A paramedian approach to the surgical level was chosen, using sequential dilators to aid in the blunt soft tissue dissection until an appropriately sized tubular retractor was inserted. The trajectory and position of the tubular retractor were then verified via a lateral fluoroscopic view before it was fixed via a table-mounted holding arm. The endoscope was then inserted, and the procedure was performed under continuous endoscopic visualization in a bimanual microsurgical fashion. A laminoforaminotomy was performed using a diamond burr and various Kerrison punches. In cases without previous cervical spine surgery, the decompression of the foramen and resection of the facet joint were not exceeded by more than 50% to avoid instability. In cases of previous fusion of the diseased segment, more than 50% of the facet can be removed. A detailed description of the surgical technique of endoscopic posterior cervical foraminotomy (EPCF) has been reported elsewhere [20,21].

The lumbar spine surgeries were also performed under general anesthesia, with the patient in a prone position. A paramedian approach with sequential dilation of the soft and muscle tissue was performed before an appropriate-sized tubular retractor was inserted and fixed in its position via a table-mounted holding arm. The endoscope was then inserted into the tubular retractor, and the procedure was performed under endoscopic visualization in a bimanual microsurgical fashion. A detailed account of the surgical technique performed for LDH, lateral recess stenosis (LRS), and lumbar synovial cyst (LSC) has been reported previously [22,23,24,25].

### 2.4. Measurement of Instrument Angulation

**Figure 2:**
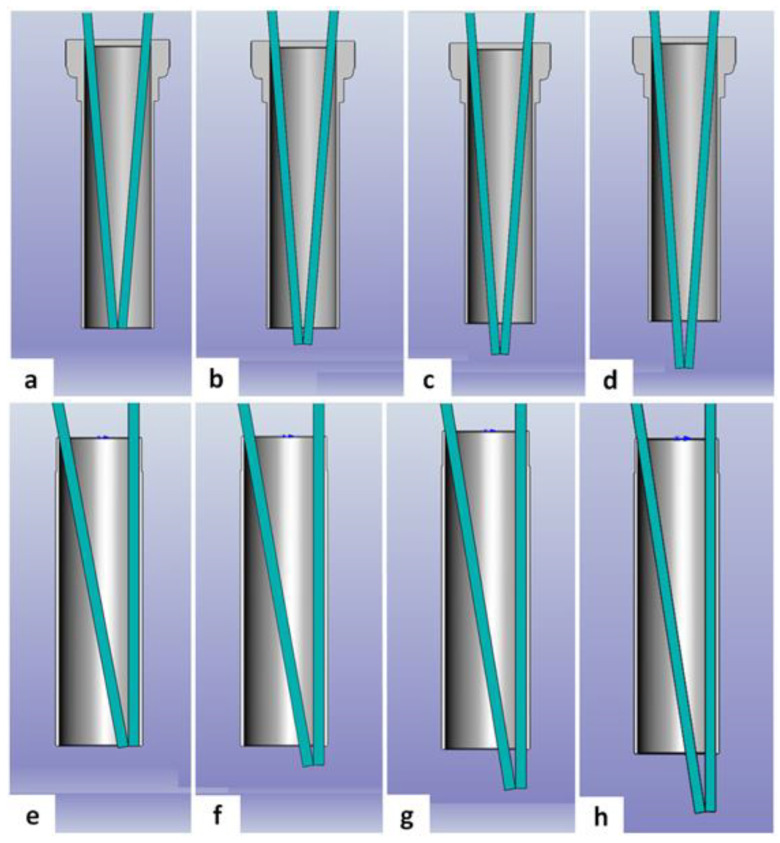
Shown is the instrument angulation simulation through the tubular retractor, demonstrating the four depths of instrument tips and two different angulations. The angulation at the center of the trocar of the first generation EasyGO™ is shown in the upper row: (**a**) 0 mm of the trocar edge, (**b**) 5 mm of the trocar edge, (**c**) 10 mm of the trocar edge, and (**d**) 15 mm of the trocar edge. The angulation at the rim of the trocar of the second generation EasyGO™ is shown in the lower row: (**e**) 0 mm of the trocar edge, (**f**) 5 mm of the trocar edge, (**g**) 10 mm of the trocar edge, and (**h**) 15 mm of the trocar edge.

The tubular retractor, the working space for instruments, and the angulation of two instruments were simulated true to scale using a 3D computer program (SolidWorks, Dassault Systems, Vélizy-Villacoublay, France). The outer diameter was set to be 3 mm for each instrument. Instrument angulation was assessed once the tips of the two instruments reached contact. For assessment, two different positions at four different depths were selected for each trocar. A total of eight positions were assessed for each tubular retractor of the first and second generations of the EasyGO™ system; see Figure 2.

### 2.5. Clinical Outcome Measures

The patients’ charts were retrospectively reviewed to extract data regarding the neurological status, pain, and functional assessment scores pre- and postoperatively. Each patient’s documents were thoroughly reviewed with a particular focus on the following parameters: intensity of arm- and leg pain, intensity of neck- and back pain using the numeric pain rating scale (NRS), presence of sensory dysfunction and presence of motor weakness according to the Janda grading [26], the Neck Disability Index (NDI) for cervical spine patients [27], and the Oswestry Disability Index (ODI) for lumbar spine patients [28]. At the final follow-up, patients underwent a physical examination. The patients’ perception of their outcome was assessed with the modified Macnab criteria [29]. In the event of a revision spinal procedure at another institution, patients were asked to present documents that were related to this repeated procedure(s) to minimize inaccurate data assessment. 

The study design was approved by the local ethical committee (149/17) and patient consent was obtained.

### 2.6. Statistical Analysis

The SPSS statistical software package (SPSS version 25, IBM, Chicago, IL, USA) was used to analyze the data. The two treatment groups for first- and second-generation EasyGO™ were analyzed with descriptive statistics. Pain level scores and clinical outcome scores were analyzed using the 2-sided Fisher exact test. A Wilcoxon test was used to compare nonparametric paired sample tests. Any *p*-values given were 2-sided; *p* > 0.05 was assumed to be sufficient to indicate statistical significance.

## 3. Results

### 3.1. Patient Population

A total of 268 consecutive patients (157 males and 117 females) were identified from our database. The mean age at the endoscopic procedure was 53.3 years (range 18–83 years).

Indications for surgery were given in patients with cervical or lumbar radiculopathy. In cases with clinical symptoms of instability, MESS was not selected as a surgical technique. Sixty-one cases of cervical spine procedures were identified. PCF was performed for osseous foraminal stenosis in 57 patients and in four patients for lateral disc herniation. One-level, two-level, and three-level cervical spine procedures were performed on 48, 12, and 1 patient each.

Two hundred and seven lumbar spine procedures were performed. Among those, 129 procedures for LDH, 61 procedures for decompression of LRS, and 17 procedures for resection of LSC were performed. One-level and two-level procedures were performed in 188 and 19 patients, respectively. A detailed compilation of operated segments and diagnosis is shown on Table 1.

### 3.2. Cervical Spine Surgery Outcomes

Preoperatively, the mean duration of symptoms was 19 weeks (1–120 weeks). Radicular arm pain was noted in 55 (90.1%). Sixty (98.4%) patients reported discomfort or neck pain. A total of 26 (42.7%) patients had a motor weakness, and 35 (57.3%) had a sensory deficit. Of note, some patients without neck and arm pain underwent the MESS procedure in the presence of a neurological deficit. 

Intraoperative: No technical complications occurred, and no conversion to an open procedure was needed. A total of 7 (11.5%) patients were operated on via a 15-mm and 54 (88.5%) patients via a 19-mm tubular retractor. The mean procedure time per segment was 60 min (20–140 min). One (1.6%) dural tear occurred, which was closed endoscopically [30]. All patients were mobilized on the day of surgery.

At discharge, 25 (41.0%) patients reported being free of arm pain, 44 (55.7%) patients reported an improvement in arm pain, and 2 (3.3%) patients reported no improvement in arm pain. A total of 14 (23.0%) patients were free of neck pain; 38 (62.3%) patients reported improvement in neck pain; and 9 (14.8%) patients had no improvement in neck pain. Among those patients with the presence of preoperative paresis, 7 (26.9%) had a complete recovery, 11 (42.3%) patients had improvement in motor strength, no improvement was noted in 6 (23.1%) patients, and 2 (7.7%) patients had a worsening of their existing preoperative paresis. A total of 13 (37.1%) patients reported a complete decline in their sensory deficit, 14 (40.0%) patients reported an improvement in their sensory deficit, and eight patients reported no change (22.9%). 

Follow-up: Fifty-two (85.2%) patients participated in the final follow-up evaluation. The mean follow-up time was 73 months (25–106). A total of 17 (32.7%) patients reported being free of neck pain, and 26 (50.0%) patients had minimal neck pain with an intensity of 1 or 2 on the NRS. At the final follow-up, 36 (69.2%) patients reported being free of arm pain. Full motor strength was found in 47 (90.4%) patients, and in 38 (73.1%) patients, no sensory deficit was noted. According to MacNab criteria, clinical success was reported in 42 (80.8%) patients.

A compilation of pain scores, functional outcome scores, and *p*-values for cervical and lumbar procedures is shown in Table 2.

### 3.3. Lumbar Spine Surgery Outcomes

The preoperative mean duration of symptoms before the endoscopic procedure was eight weeks (range 1 day–120 weeks). Radicular leg pain was noted in 187 (90.3%) patients. One hundred eighty-five (89.4%) patients reported discomfort or back pain. One hundred and nineteen (57.5%) patients had a motor weakness, and 117 (55.6%) patients had a sensory deficit. Of note, some patients without back and leg pain underwent the MESS procedure in the presence of a neurological deficit. 

Intraoperative: No emergencies or complications requiring conversion from microsurgery to open surgery occurred using the endoscopic system. One patient needed a switch from a 15-mm to a 19-mm tubular retractor due to limited instrument angulation caused by obesity. Forty-four (21.3%) patients were operated on via a 15-mm trocar and 163 (78.7%) patients via a 19-mm trocar. The mean surgical time was 60 min (24–189 min). An incidental dural tear was noted in nine (6.9%) LDH cases, six (9.8%) LRS cases, and four (23.5%) LSC cases. The overall dural tear rate was 9.1%. The tear of the dura was small in all cases without severe herniation of nerve roots. Therefore, in all cases, the dural tear was repaired endoscopically using an autologous muscle graft and a fibrin-sealed collagen sponge. A detailed account of the technique for endoscopic dural tear closure has been reported elsewhere [30]. All patients were mobilized on the day of surgery. 

At discharge, 97 (46.9%) patients reported being free of leg pain; 106 (51.2%) patients reported an improvement of leg pain; 3 (1.4%) patients reported no improvement of leg pain; and 1 patient (0.5%) reported a worsening of leg pain. A total of 88 (42.5%) patients were free of back pain; 105 (50.7%) patients reported improvement in back pain; and 11 (5.3%) patients had no improvement in back pain. Among those patients with the presence of preoperative paresis, 26 (21.8%) patients had a complete recovery, 69 (58.0%) patients had improvement in motor strength, no improvement was noted in 14 patients (11.8%), and no worsening of paresis was noted. In 2 (1.7%) patients, a new temporary paresis (4/5, according to Janda) was recorded. Sixty-two (53.0%) patients reported a complete decline in their sensory deficit, 39 (33.3%) patients reported an improvement in their sensory deficit, and 16 patients reported no change (13.7%).

One hundred seventy-seven (85.5%) patients participated in the final follow-up evaluation. The mean follow-up time was 62 months (25–102). At the final follow-up, 136 (76.8%) patients reported being free of leg pain, and 40 (22.6%) reported minimal leg pain with an intensity of 1 or 2 on the NRS. 

At the final follow-up, 122 (68.9%) patients reported being free of back pain, and 22 (12.4%) reported minimal back pain with an intensity of 1 or 2 on the NRS. In 147 (83.1%) patients with full motor strength, 23 (13.0%) had improved motor strength compared to their preoperative status. No improvement was noted in 5 (2.8%) patients; in 2 patients (1.1%), a new mild paresis was stressed. In 153 (86.4%) patients, no sensory deficit was noted, and a residual sensory deficit was reported in 24 (13.6%) patients. 

According to Macnab’s criteria, clinical success was noted in 155 (87.6%) patients. A compilation of pain scores, functional outcome scores, and *p*-values for cervical and lumbar procedures is shown in Table 3.

### 3.4. Revision Surgeries

Cervical spine: In 11 (18.0%) cases, a repeated procedure was performed. Nine procedures were performed within the first year, and seven repeated procedures were performed at the index segment. 

Repeated procedures were performed for the evacuation of an epidural hematoma in one patient. Anterior cervical fusion was performed in seven patients. EPCF was performed in two patients and open microsurgical PCF in one patient. 

Lumbar spine: In 28 (13.5%) cases, a repeated procedure was performed. Fifteen repeated procedures were performed within the first year. In 20 (11.3%) cases, repeated procedures were performed at the index segment. In one (0.5%) patient each, a repeated procedure was performed to evacuate an epidural hematoma, nerve root adhesiolysis, microsurgical cyst resection and dural repair, kyphoplasty for osteoporotic compression fracture, and endoscopic denervation of the facet joints. 

In 11 (5.3%) cases, a microsurgical discectomy was performed at the index segment and in 6 cases at the adjacent segment. In 10 out of 129 (7.7%) cases, a true recurrent LDH was the cause of repeated procedures. In two cases, repeated decompression of the index and the adjacent segment was performed. In two (1.0%) patients, a transforaminal lumbar interbody fusion (TLIF) for degenerative spondylolisthesis was performed.

Clinical success was 52.0% for patients who underwent a repeated procedure at the index segment and 68.7% for patients who underwent a repeated procedure at the adjacent segments.

### 3.5. Assessment of Instrument Angulation 

The 15-mm trocar was not produced in 90-mm length for the first generation of the EasyGO™ system. Therefore, 64 different instrument angulations were measured for the first generation of the EasyGO™ system. Seventy-two different instrument angulations were calculated for the second generation of the EasyGO™ system. The comparison of instrument angulation for all matches concerning outer diameter and trocar length showed greater values in any position for the second generation of the EasyGO™ system. The difference for each comparison decreases as the length of the trocar increases, and the difference is also reduced as the depth of the instrument tip increases. A detailed compilation of all instrument angulations and differences is shown in Table 4.

### 3.6. Comparison of MESS Systems

The surgical time for cervical and lumbar procedures and the repeated procedure rate were statistically significantly shorter using the second generation of the EasyGO system. No statistically significant differences were noted for compilations or clinical success. A detailed compilation of all comparisons, including the *p*-values, is shown in Table 5.

## 4. Discussion

Microsurgical techniques were introduced in the late 1970s and achieved a good clinical outcome in long-term follow-up studies [3,31]. The principal idea of minimally invasive spine surgery (MISS) is the reduction of soft tissue and muscle trauma while approaching the spine, while the treatment goal is analogous to that of the open technique. Many MISS studies have been performed to evaluate the clinical outcome for the treatment of cervical foraminal stenosis, lumbar disc herniation, lumbar lateral recess stenosis, and synovial cyst [32,33,34,35,36,37,38]. Advantages of MISS include a lower postoperative requirement for pain medication, less blood loss, earlier mobilization, and a short hospitalization [15].

However, higher rates of nerve root injury, recurrent LDH rate, and incidental durotomy compared to microsurgical techniques have also been reported. In the authors’ opinion, it is challenging to assess the safety, success, and reliability of a surgical technique even in prospective controlled trials because the final result of the procedure is influenced by the surgeon’s personality structure, risk aversion, experience in microsurgical procedures, the complexity of the patient’s condition, open-mindedness regarding new surgical techniques, and the success of the initial procedures.

Limited visualization, limited instrument angulation, and the learning curve have been used to explain complications in endoscopic or MISS techniques [8]. In contrast to the abovementioned concerns, the literature shows that high-definition microendoscopic visualization is superior to microscopic visualization in tubular-assisted procedures [12]. Additionally, the learning curve for microendoscopic procedures reaches a plateau after only 10–30 procedures for experienced microsurgical spine surgeons regarding surgical time and intraoperative complications [39,40]. To the best of the authors’ knowledge, no other study has been published that assessed the influence of instrument angulation on clinical outcome, complications, and perioperative parameters. The results of the present study demonstrate that an increase of 14% to 18% in the tubular workspace has significantly reduced the overall surgical time in cervical and lumbar procedures, even in very experienced MESS surgeons. However, there was no influence on the clinical success (i.e., Macnab’s criteria), rate of repeated procedures, or complications. The overall results of instrument angulation show that the second generation achieves almost the same degree of instrument angulation via a tubular retractor, whose outer diameter is 4 mm smaller than the first generation. It remains to be seen if the increased workspace influences the learning curve. The present study’s authors had several personal communications with spine surgeons at other institutions who used both generations of this system. These surgeons have consistently reported that increased angulation positively affected their surgical workflow.

The present study’s mean surgical time was 60 min shorter than other studies of open and tubular-assisted PCF [41]. In the current series, 80% of patients reported clinical success after EPCF, comparable to 87% improvement of symptoms in open PCF [42]. Evaniew et al. reported a higher re-operation rate after minimally invasive spine surgery. In the authors’ opinion, those results should be interpreted with caution because four out of five included studies compared full endoscopic and microendoscopic PCF to anterior cervical decompression and fusion procedures. The revision surgery rate of 13–18% following open PCF within 2–10 years and 24% after more than ten years of follow-up has been reported [42,43]. The repeat procedure rate of the present study is, therefore, comparable to the rates of open PCF. The mean duration from the initial to the repeated procedure of the index level differs from 5 to 55 months with the microendoscopic PCF studies [44].

Comparison studies of microendoscopic and microsurgical procedures are critical because the surgeon’s skills and patient selection mainly influence the procedure’s results. Therefore, the study results differ considerably, as German et al. reported a mean surgical procedure time for tubular assisted discectomy of about 160 min [16], which is twice as long compared to the mean of 60 min in the present study and reported data from other microendoscopic studies [16,45,46,47,48]. The rate of intraoperative dural tears in the present study is in line with other MISS studies [46,48] and similar to microsurgical procedures of 2.8% to 5.6% [46,48,49]. The clinical success rate of the present study is corroborated by other long-term follow-up studies, which have reported a clinical success rate for microendoscopic discectomy of 74–97% [47,50]. This rate is similar to the rate of 80% satisfaction reported after two and eight years following open microdiscectomy [49,51].

A significant concern for the treatment of LDH, is the reoccurrence of LDH which might result in a repeated procedure. Evaniew et al. stated in their meta-analysis that MISS procedures should not be used routinely to treat cervical and lumbar disc herniations due to unfavorable risk-benefit ratios, higher rates of nerve root injury, dural tear, and repeated procedures compared to open procedures. A closer look at this meta-analysis revealed that Evaniew et al. included a variety of surgical techniques under the generic term of MISS, such as full endoscopic techniques, microendoscopic techniques, and tubular assisted techniques. Surgical indications for those techniques might vary, and a comparison is of limited validity. Limited instrument angulation, the difficulty of tissue manipulation, and poor surgical field visualization have been considered causes of this undesirable event in tubular assisted procedures [18]. The rate of repeated procedures for recurrent LDH varies from 2.1 to 11.7% following MESS, with an average period of 14 to 39 months from the initial MESS to the repeated procedure [46,47,48,52]. The present study’s results align with the studies mentioned above regarding the repeated procedure rate for recurrent LDH (i.e., 7.7%) and the mean period from initial to repeated procedure (i.e., 11 months). Those results are also comparable to open microsurgical discectomy, with reported rates of 8.2% to 9.1% [31,53].

The above-mentioned studies corroborate that MESS techniques should be considered equal to the so-called “microsurgical gold standard” or even superior. The MESS approach for the treatment of lumbar disc herniation located within the spinal canal is, in general, slightly more lateral to the midline compared to the microsurgical approach. In contrast to the open microsurgical approach, the muscle tissue is dilated, and the multifidus muscle insertion is not dissected. If this technical nuance and preservation influence index and adjacent segment degeneration still need to be clarified, Li et al. reported a reduced rate of adjacent segment degeneration for minimally invasive fusion procedures [54]. To the best of the authors’ knowledge, no comparison study exists that addresses this topic in the long term for the treatment of lumbar disc herniation. According to the data of the SPORTS trial, the repeated procedure rate increased from 11% at five-year follow-ups to 15% at eight years of follow-up, whereas 9% were performed for recurrent LDH [49,53]. In the present study, the repeated procedure rate following MESS for LDH was 14.7%, and the rate for recurrent LDH was 7.7%. Aihara et al. reported similar rates for overall repeated procedures (i.e., 12.4%) and for recurrent LDH (i.e., 7.7%), and this rate remained stable up to ten-year follow-up [52,55]. The body of literature shows that MESS should be considered at least equal to open microsurgical procedures because of the positive effects in the early postoperative periods, the long-term clinical outcome, and the long-term repeat procedure rates for cervical and lumbar disorders.

## 5. Conclusions

MESS achieves high clinical success rates and reduced pain levels for treating degenerative cervical and lumbar spine procedures. The overall rate of repeated procedures for recurrent LDH was 7.7%. Increased tubular working space with improved instrument angulation reduces overall surgical time and revision lumbar surgery rates at the index level.

## Figures and Tables

**Table 1:** Compilation of diagnosis and operating level.

		Osseous Foraminal Stenosis	Lateral Disc Herniation			Disc Herniation	Lateral Recess Stenosis	Synovial Cyst
**Cervical Segment**	C4/5	1		**Lumbar Segment**	L1/2	1		
	C5/6	9 *			L2/3	9	1	1
	C6/7	27 *	3		L3/4	13	3	1
	C7/T1	7	1		L4/5	50	26	11
					L5/S1	50	18	4
	C4–C6	2						
	C5–C7	6			L3–L5	1	5	
	C6–T1	4			L4–S1	5	8	
						
	C3–C6	1 **						

* patients with bilateral PCF. ** patients with bilateral PCF at the C4–5 level.

**Table 2:** Compilation of clinical outcomes in EPCF.

Osseous Foraminal Stenosis and Lateral Disc Herniation	Preoperative	Postoperative	*p*-Value	Follow-Up	*p*-Value
**Mean arm pain**	6.2 (range: 2–10)	1.5 (range: 0–6)	<0.001	1.1 (range:1–8)	<0.001
**Mean neck pain**	4.0 (range: 1–10)	2.2 (range: 0–10)	<0.001	1.9 (range: 0–10)	<0.001
**Mean Neck Disability** **Index (NDI)**	27% (range: 0–70%)	14% (range: 0–70%)	<0.001	10% (range 0–72%)	<0.001

**Table 3:** Compilation of clinical outcomes for lumbar MESS.

**Diagnosis**	**Preoperative Leg Pain**	**Postoperative Leg Pain**	***p*-Value**	**Follow-Up Leg Pain**	***p*-Value**
Disc herniation	7.2 (range: 0–10)	1.2 (range: 0–7)	<0.001	0.5 (range: 0–5)	<0.001
Lateral recess stenosis	6.2 (range: 0–10)	1.4 (range: 0–8)	<0.001	0.9 (range: 0–7)	<0.001
Synovial cyst	6.9 (range: 0–9)	1.8 (range: 0–4)	<0.001	0.7 (range: 0–6)	<0.001
**Diagnosis**	**Preoperative Back Pain**	**Postoperative Back Pain**	***p*-Value**	**Follow-Up Back Pain**	***p*-Value**
Disc herniation	2.7 (range: 0–10)	1.6 (range: 0–7)	<0.001	1.0 (range: 0–8)	<0.001
Lateral recess stenosis	6.6 (range: 0–10)	1.7 (range: 0–9)	<0.001	1.8 (range: 0–6)	<0.001
Synovial cyst	4.8 (range: 0–9)	1.8 (range: 0–4)	0.001	2.0 (range: 0–5)	0.005
**Diagnosis**	**Preoperative Oswestry Disability Index**	**Postoperative Oswestry Disability Index**	***p*-Value**	**Follow-Up Oswestry Disability Index**	***p*-Value**
Disc herniation	21 (range: 0–50)	9 (range: 0–66)	<0.001	11 (range: 0–64)	<0.001
Lateral recess stenosis	26 (range: 0–60)	11 (range: 0–36)	<0.001	15 (range: 0–60)	<0.001
Synovial cyst	29 (range: 0–66)	15 (range: 0–36)	0.011	16 (range: 0–44)	0.013

**Table 4:** Instrument angulation.

			1. Generation		2. Generation		1. vs. 2. Generation	
Trocar Diameter (mm)	Length (mm)	Instrument Depth (mm)	Instrument Angel at the Center of the Trocar (°)	Instrument Angel at the Inner Rim of the Trocar (°)	Instrument Angel at the Center of the Trocar (°)	Instrument Angel at the Inner Rim of the Trocar (°)	Difference of Angle at the Center (°)	Difference of Angle at the Inner Rim (°)
15	40	0	6.37	6.92	9.18	9.14	2.81	2.22
15	40	5	5.88	6.39	8.28	8.26	2.40	1.87
15	40	10	5.46	5.93	7.55	7.53	2.09	1.60
15	40	15	5.11	5.54	7.00	6.95	1.89	1.41
15	70	0	4.67	4.66	5.56	5.56	0.89	0.90
15	70	5	4.42	4.41	5.22	5.22	0.80	0.81
15	70	10	4.16	4.19	4.92	4.91	0.76	0.72
15	70	15	3.96	4.00	4.66	4.63	0.70	0.63
15	90	0	N/A	N/A	4.38	4.38	N/A	N/A
15	90	5	N/A	N/A	4.17	4.16	N/A	N/A
15	90	10	N/A	N/A	3.97	3.97	N/A	N/A
15	90	15	N/A	N/A	3.78	3.81	N/A	N/A
19	40	0	10.92	10.89	13.40	13.26	2.48	2.37
19	40	5	10.08	10.07	12.17	12.06	2.09	1.99
19	40	10	9.36	9.35	11.14	11.06	1.78	1.71
19	40	15	8.76	8.73	10.31	10.26	1.55	1.53
19	70	0	7.28	7.28	8.31	8.27	1.03	0.99
19	70	5	6.89	6.90	7.81	7.79	0.92	0.89
19	70	10	6.55	6.56	7.38	7.35	0.83	0.79
19	70	15	6.26	6.26	7.02	6.95	0.76	0.69
19	90	0	5.56	5.57	6.59	6.58	1.03	1.01
19	90	5	5.33	5.35	6.28	6.27	0.95	0.92
19	90	10	5.12	5.14	5.99	5.98	0.87	0.84
19	90	15	4.83	4.94	5.72	5.70	0.89	0.76
23	40	0	14.73	14.58	17.12	16.81	2.39	2.23
23	40	5	13.60	13.49	15.62	15.38	2.02	1.89
23	40	10	12.63	12.54	14.36	14.18	1.73	1.64
23	40	15	11.82	11.75	13.34	13.21	1.52	1.46
23	70	0	9.61	9.58	10.90	10.81	1.29	1.23
23	70	5	9.11	9.09	10.27	10.20	1.16	1.11
23	70	10	8.66	8.65	9.71	9.65	1.05	1.00
23	70	15	8.26	8.26	9.22	9.16	0.96	0.90
23	90	0	7.40	7.50	8.66	8.62	1.26	1.22
23	90	5	7.10	7.20	8.25	8.22	1.15	1.12
23	90	10	6.83	6.83	7.89	7.86	1.06	1.03
23	90	15	6.59	6.59	7.58	7.54	0.99	0.95

**Table 5:** Clinical outcome comparison: 1. vs. 2. generation MESS system.

	1. Generation	2. Generation	*p*-Value
**Overall clinical success**	83.6%	88.5%	0.342
**Clinical success cervical spine**	75.8%	100%	0.318
**Clinical success lumbar spine**	85.5%	89.1%	0.498
**Overall repeated procedure**	22.4%	10.8%	**0.012**
**Repeated procedure cervical spine**	27.3%	12.5%	0.483
**Repeated procedures lumbar spine**	22.4%	8.9%	**0.017**
**Complication cervical procedures**	1 (2.5%)	0 (0%)	1.000
**Complication lumbar procedures**	6 (6.7%)	13 (11.0%)	0.210
**Overall surgical time cervical procedure**	67 min	47 min	**>0.001**
**Surgical time of cervical procedure via 19-mm trocar**	67 min	53 min	**0.017**
**Overall surgical time lumbar procedure**	68 min	55 min	**0.002**
**Surgical time of lumbar DH procedure via 15-mm trocar**	57 min	50 min	0.068
**Surgical time of lumbar DH procedure via 19-mm trocar**	77 min	52 min	**>0.001**
**Surgical time of lumbar LRS procedure via 15-mm trocar**	42 min	52 min	0.476
**Surgical time of lumbar LRS procedure via 19-mm trocar**	59 min	46 min	0.088

## Data Availability

The data are unavailable due to privacy and ethical restrictions.
